# Current advisory interventions for grazing ruminant farming cannot close exceedance of modern background sediment loss – Assessment using an instrumented farm platform and modelled scaling out

**DOI:** 10.1016/j.envsci.2020.11.004

**Published:** 2021-02

**Authors:** A.L. Collins, Y. Zhang, H.R. Upadhayay, S. Pulley, S.J. Granger, P. Harris, H. Sint, B. Griffith

**Affiliations:** Sustainable Agriculture Sciences, Rothamsted Research, North Wyke, Okehampton, Devon, EX20 2SB, UK

**Keywords:** Soil erosion, Sediment, Ruminant farming, Mitigation

## Abstract

Water quality impairment by elevated sediment loss is a pervasive problem for global water resources. Sediment management targets identify exceedance or the sediment loss ‘gap’ requiring mitigation. In the UK, palaeo-limnological reconstruction of sediment loss during the 100–150 years pre-dating the post-World War II intensification of agriculture, has identified management targets (0.20−0.35 t ha^−1^ yr^−1^) representing ‘modern background sediment delivery to rivers’. To assess exceedance on land for grazing ruminant farming, an integrated approach combined new mechanistic evidence from a heavily-instrumented experimental farm platform and a scaling out framework of modelled commercial grazing ruminant farms in similar environmental settings. Monitoring (2012–2016) on the instrumented farm platform returned sediment loss ranges of 0.11−0.14 t ha^−1^ yr^−1^ and 0.21−0.25 t ha^−1^ yr^-1^ on permanent pasture, compared with between 0.19−0.23 t ha^−1^ yr^-1^ and 0.43−0.50 t ha^−1^ yr^−1^and 0.10−0.13 t ha^−1^ yr^−1^and 0.25−0.30 t ha^−1^ yr^-1^ on pasture with scheduled plough and reseeds. Excess sediment loss existed on all three farm platform treatments but was more extensive on the two treatments with scheduled plough and reseeds. Excessive sediment loss from land used by grazing ruminant farming more strategically across England, was estimated to be up to >0.2 t ha^−1^ yr^−1^. Modelled scenarios of alternative farming futures, based on either increased uptake of interventions typically recommended by visual farm audits, or interventions selected using new mechanistic understanding for sediment loss from the instrumented farm platform, returned minimum sediment loss reductions. On the farm platform these were 2.1 % (up to 0.007 t ha^−1^ yr^−1^) and 5.1 % (up to 0.018 t ha^−1^ yr-1). More strategically, these were up to 2.8 % (0.014 t ha^−1^ yr^−1^) and 4.1 % (0.023 t ha^−1^ yr^−1^). Conventional on-farm measures will therefore not fully mitigate the sediment loss gap, meaning that more severe land cover change is required.

## Highlights

• Excess sediment loss from land used by grazing ruminant farms assessed.
• Modern background sediment loss rates of 0.20−0.35 t ha^−1^ yr^−1^ used as targets.
• Instrumented farm platform data suggested losses up to 0.50 t ha^−1^ yr^−1^.
• Modelled scaling out returned exceedances of up to >0.20 t ha^−1^ yr^−1^.
• Current advisory measures cannot reduce sediment loss to the target rates.

## Graphical abstract

**Figure:**
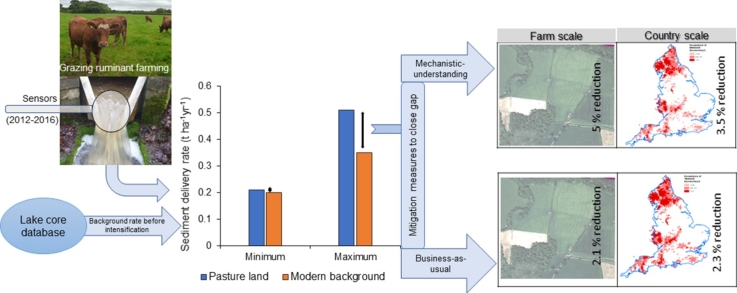

## 1 Introduction

Improved pasture and rough grazing accounts for ∼67 % of the agricultural land area of the UK (Defra, 2016), supporting production with a net worth of ∼£8 billion to the UK economy (Orr et al., 2016). Well-managed grazing can support important ecosystem goods or services including water infiltration, purification or storage, erosion prevention, nutrient cycling, pollination, biodiversity, biomass production, carbon storage and sequestration and flood reduction (Pykala, 2000; Rook and Tallowin, 2003; Villoslada et al., 2019). Intensive ruminant farming, however, runs the risk of excessive defoliation and soil compaction, thereby increasing exposure of bare soil to erosive agents (Edmond, 1958; Matches, 1992) and running the risk of environmental degradation (Mulholland and Fullen, 1991; Trimble and Mendel, 1995; Evans, 1998; Reynolds et al., 2002). Although much of the work in the UK investigating soil erosion on agricultural land and, its on-site and off-site consequences, originally focused on arable production systems (Evans, 1971, 1990), since the late 1990′s, there has been growing recognition of the important role of modern intensive ruminant farming in generating soil erosion and sediment problems (Collins et al., 1997; Evans, 1998; Walling, 2005; Bilotta et al., 2008; Granger et al., 2010; Peukert et al., 2014). At the same time, the need for sustainable intensification of modern agriculture is being debated in the context of the need to meet rising demand for food given global population growth and changing dietary patterns, whilst preserving ecosystem services (Godfray and Garnett, 2014; Balmford et al., 2018). The environmental impact of agriculture, including externalities related to soil erosion and sediment loss, is therefore under continued scrutiny (Montgomery, 2007; FAO, 2011; Whitecraft and Huggins, 2013; Montanarella, 2015; Borrelli et al., 2017).

Whilst soil loss can reduce fertility and crop yields (Pimental and Burgess, 2013), much focus in terms of the impact of agriculture on this component of the environmental pillar of sustainable intensification, has been directed towards off-site consequences for sediment-related water quality. Both internationally and in the UK, elevated levels of fine-grained (<2 mm) sediment represent one of the primary and most pervasive causes of water quality impairment (Easterling et al., 2000; Gray, 2008; Wharton et al., 2017; Wilkes et al., 2019). Fine sediment redistribution exerts an important control on the transfers and fate of nutrients and contaminants (Horowitz, 2008; Girmay et al., 2009; Herrero et al., 2018). Through top-down abiotic factors, excess fine-grained sediment can result in detrimental impacts at organism and community scales at all trophic levels (Kemp et al., 2011; Jones et al., 2012a, b, Jones et al., 2014). Given such problems, corresponding water quality guidelines and thresholds have been established around the world using either substrate, or more commonly, water column metrics (Collins et al., 2011). The former focus on substrate composition or riffle stability (Kondolf, 2000; Kappeser, 2002), whereas the latter use light penetration, turbidity or sediment concentration thresholds or summary statistics (Wilber, 1983; USEPA, 2007; Thompson et al., 2014).

Despite the proposal of various water column guidelines and thresholds for sediment in lotic receptors, there remains, however, a poor understanding of, and lack of consensus on, the targets that policy teams and catchment managers should be setting for compliance. In Europe and the UK, sediment management targets are not well developed with the result that until its repeal in 2013, the guideline annual average suspended sediment concentration of 25 mg L^−1^ set by the Freshwater Fish Directive (FFD; 78/659/EC) was used to assess suspended sediment compliance in rivers (Collins and Anthony, 2008). Using a single blanket target in disregard of heterogeneous environmental settings and management characteristics has, however, been criticized with the result that alternative approaches to evaluating sediment compliance have been proposed and tested. Some work in the UK has, for example, examined scope for using routine but infrequent strategic datasets as a basis for establishing five mean background suspended sediment concentration ranges (Bilotta et al., 2012) or higher resolution monitoring as a basis for understanding spatial and temporal variations in concentrations at reference sites (Grove et al., 2015). Alternatively, sediment pressure biomonitoring tools have been developed to assess sediment pollution in the aquatic environment (Murphy et al., 2015; Turley et al., 2016).

Despite the focus of water quality policy in the UK on achieving good ecological status since the introduction of the Water Framework Directive in 2000 (European Union, 2000), determining the biological relevance of conventional sediment monitoring data continues to pose a scientific challenge (Chapman et al., 2014). Regime-based (e.g. area normalized sediment yields) sediment targets therefore offer a pragmatic alternative for assessing sediment compliance and any associated ‘gap’ (Collins et al., 2011; Foster et al., 2011). Sediment regime data integrate the effects of both intrinsic and anthropogenic controls and can be generated using either contemporary (Cooper et al., 2008) or reconstructed historical data (Rose et al., 2011). Accordingly, the work reported herein adopted this type of management target and examined suspended sediment loss from grazing ruminant farming in England under business-as-usual and future alternative management scenarios. The aim was to quantify any excess sediment loss and the technically feasible reductions in the sediment loss ‘gap’ under alternative management futures. The management scenarios were designed to embody current on-farm advice as directed by visual farm inspections and audits, and an alternative mechanistically-based scenario using evidence from the world’s most instrumented grazing ruminant farm platform in SW England.

## 2 Materials and methods

### 2.1 An integrated monitoring and modelling approach

The technically feasible scope for closing any sediment loss ‘gap’ associated with the use of land for modern grazing ruminant farming was assessed using the integration of empirical evidence and modelling. In so doing, the intention was to scale out beyond the heavily-instrumented farm platform with extensive empirical datasets to modelled farms in the same sector and in matching environmental settings, to demonstrate the relevance of science and associated findings from the instrumented platform to farmers across England. In short, the integrated approach comprised: (i) selection of a management target for assessing exceedance (i.e. the sediment loss ‘gap’ requiring mitigation) under business-as-usual grazing ruminant farming; (ii) use of a heavily-instrumented farm platform to assess the sediment ‘gap’ empirically, under current best management, and to provide mechanistic information on soil loss and sediment delivery for helping to select appropriate on-farm interventions for mitigating the ‘gap’; (iii) modelling of the technically feasible reductions in the sediment loss ‘gap’ using different management scenarios on the heavily-instrumented farm platform, and; (iv) modelling of the technically feasible reductions in the sediment loss ‘gap’ using a comparison of the same management scenarios on representative model grazing ruminant farms in similar environmental settings across England.

### 2.2 Selection of the management target for assessing the sediment loss ‘gap’ on land used by grazing ruminant farming

Any exercise quantifying a water pollution (e.g., sediment) ‘gap’, and the corresponding scope for reducing that externality, requires the selection of a meaningful management target. Whereas the notion of reducing any excess sediment loss associated with modern farming to truly ‘intrinsic’ levels has been mooted, society requires our land to produce food alongside other goods and services. In this context, it is unrealistic to strive for ‘intrinsic’ rates of sediment loss and alternative targets must be adopted. Palaeo-environmental reconstruction (e.g., Edwards and Whittington, 2001; Macklin et al., 2010) data from UK lakes has been used to estimate more realistic targets based on ‘modern background’ sediment loss (Foster et al., 2011). The logic here, is that since the most recent substantial increase in UK sediment loss occurred in tandem with post-WW II intensification (Rose et al., 2011), the last ∼100–150 years of landscape sediment response archived in dated lake sediment profiles can be used to establish provisional ‘modern background’ management targets. These targets represent sediment loss in the period immediately pre-dating post-WW II intensification. Accordingly, Table 1 presents estimates of ‘modern background sediment delivery to rivers’ (MBSDR) for lowland agricultural land across England and Wales (Foster et al., 2011) wherein, dated lake profiles were matched to dominant land cover types in the contributing catchments. The targets were assumed to represent sediment delivery from different dominant land cover classes to rivers, rather than net specific sediment yields, since correction of the former to represent the latter, would require reliable data for long-term sediment retention in landscape stores (e.g., floodplains) across scales. Two classes of MBSDR were proposed by Foster et al. (2011) to represent uncertainty: a ‘target modern background sediment delivery to rivers’ (TMBSDR), and; a ‘maximum modern background sediment delivery to rivers’ (MMBSDR) (Table 1). Since the environmental setting at the NWFP bridges categories A and B in Table 1, a catchment on the farm platform was deemed in exceedance of MBSDR if both the lower and upper 95 % confidence limits of measured annual specific sediment loss (t ha^−1^ yr^−1^) exceeded the corresponding upper thresholds (i.e., 0.2 and 0.35 t ha^−1^ yr^−1^; Table 1) from Foster et al. (2011).

**Table 1:** Estimates of TMBSDR and MMBSDR for lowland agricultural land across England (Foster et al., 2011).

Land cover	TMBSDR (t ha^−1^ yr^−1^)	MMBSDR(t ha^−1^ yr^−1^)
Lowland agriculture (A)^a^	<0.1	0.15
Lowland agriculture (B)^b^	<0.2	0.35

a Group A agricultural catchments are those with soils at very low or low-moderate water erosion risk based on the classification of Evans (1990) and/or are less heavily dissected (<3°).

b Group B agricultural catchments are those with soils at high to very high water erosion risk based on the classification of Evans (1990) and/or are more heavily dissected (slopes >3°).

### 2.3 Use of the instrumented North Wyke Farm Platform to measure the sediment loss ‘gap’ from land used for grazing ruminant farming

**Fig. 1:**
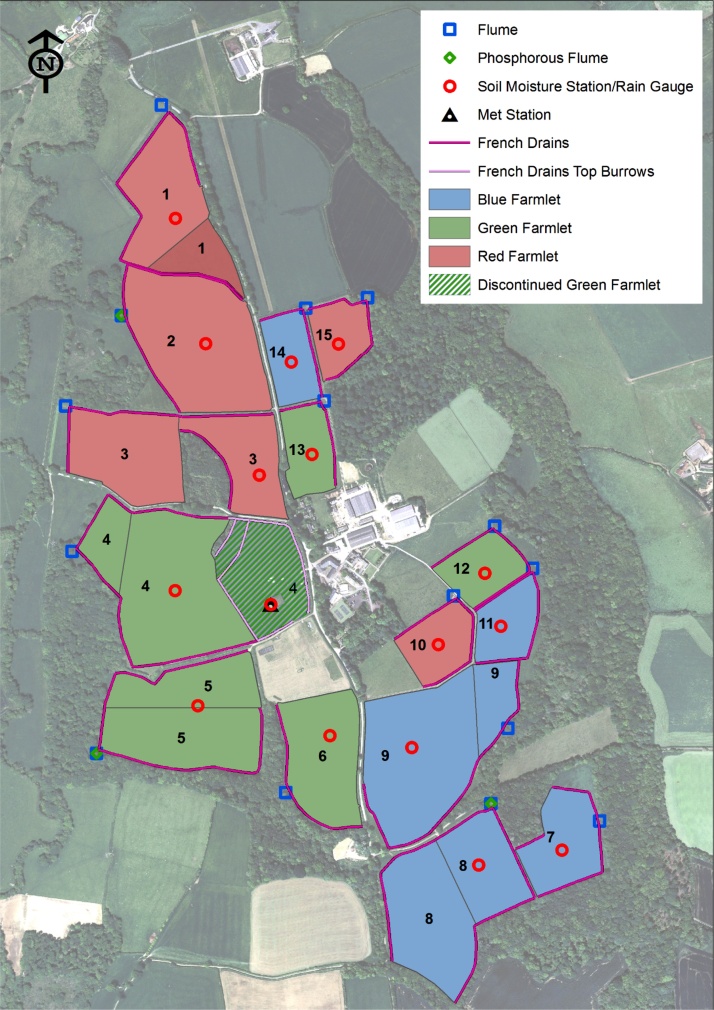
Design of the NWFP. Numerals correspond to catchment numbers: Green farmlet - catchments 4, 5, 6, 12 and 13; Blue farmlet - catchments 7, 8, 9, 11 and 14; Red farmlet - catchments 1, 2, 3, 10 and 15.

The North Wyke Farm Platform (NWFP; 50^∘^46′10′′N, 3^∘^54′05′′W; Orr et al., 2016) is a UK National Capability for research that was established in 2010 for systems scale analysis of the sustainability of lowland ruminant (beef and sheep) production systems. It comprises three farmlets (21 ha each), each of which have five catchments (1.62–8.08 ha) (Fig. 1). Each catchment is hydrologically-isolated using the combined effect of topography, impermeable subsoils and 9.2 km of French drains (800-mm deep trenches with a perforated drainage pipe backfilled to the surface with 20–50 mm clean granite, carbonate-free, stone chips) bordering the catchments. Six of the 15 catchments have field divisions providing 21 fields in total. Soils are dominated by the Hallsworth (Dystric Gleysol) and Halstow (Gleyic Cambisol) (Avery, 1980) series; slightly stony clay loam topsoil (approximately 36 % clay) overlying a mottled stony clay (approximately 60 % clay), derived from underlying Carboniferous culm rocks (Harrod and Hogan, 2008). Since the subsoil is impermeable to water and is seasonally waterlogged, excess water moves by surface and sub-surface lateral flow to the French drains at the edge of the fields. The outfalls of the 15 catchments are each equipped with a flume laboratory consisting of H-flumes (TRACOM Inc., Georgia, USA; capacity for a 1 in 50-year storm event) and pressure transducers (OTT hydromet, Loveland, CO., USA) for gauging stage height and discharge, multi-parameter sondes (YSI 6600, Xylem Inc Rye Brook, New York, U.S; turbidity, dissolved oxygen, conductivity, pH, temperature, ammonium, ammonia) and an automatic water sampler (ISCO 3700, Teledyne ISCO). The sondes are installed in stainless steel by-pass flow cells to accommodate the discontinuous field runoff driven by prevailing soil moisture conditions and the associated vulnerability to technical issues arising from frequent drying out. Discharge and water quality parameters are recorded at 15-minute intervals. Automatic water samples are collected on a campaign basis and returned to the laboratory for processing.

**Fig. 2:**
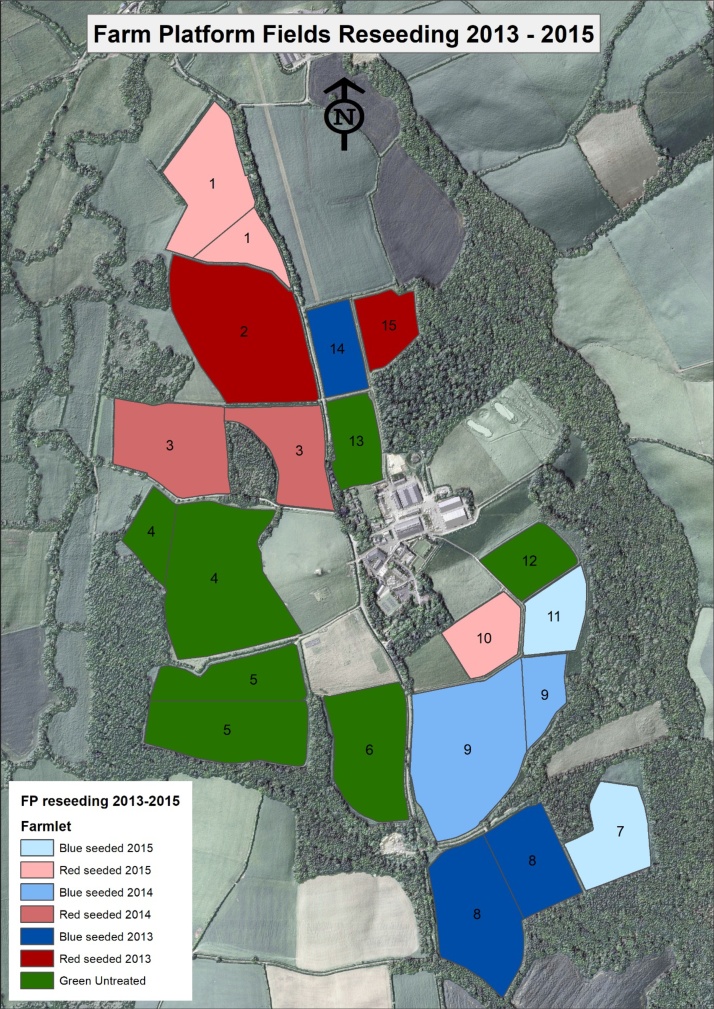
Reseeds (2013-2015, inclusive), by experimental farmlet, on the NWFP.

The three original farmlets of the NWFP were established to compare the dominant lowland grazing land covers and associated management strategies in the UK; long-term permanent pasture (green farmlet; perennial ryegrass; *Lolium perenne* L.), grass-clover mixes (blue farmlet; using white clover; *Trifolium repens* L. cv AberHerald) for nitrogen fixation (Elgersma and Hassink, 1997) and associated reductions in artificial fertiliser inputs (none received 2013−15), and planned re-seeding with a high sugar perennial ryegrass monoculture (red farmlet; cv. Abermagic). At the outset of the 2012–2013 water year, all catchments were under long-term permanent pasture, but during the summers of 2013, 2014 and 2015, fields on the blue and red farmlets were progressively ploughed and reseeded (see schedule in Fig. 2) to introduce the new grass swards. Here, target catchments were sprayed with glyphosate to kill the existing grass, followed by ploughing and re-seeding (see management details in Table S1).

Grazing management on the NWFP is designed to illustrate best practice. In autumn, 30 (originally Charolais × Hereford-Friesian, but now Stabiliser) calves at the point of weaning are randomly assigned to each farmlet from an adjacent cow-calf enterprise. Cattle are housed from typically October to April to avoid soil structural degradation, then kept outdoors on their respective farmlet until they reach target weights of around 555 kg for heifers and 620 kg for steers. Farmyard manure from the housing period is stored in middens until pastures are ready for fertilisation between silage cuts. In the case of the sheep, 50 Suffolk × Mule ewes and their lambs sired by Charollais rams are randomly assigned to each farmlet each spring. Given the lambing rate of 1.8, this results in a flock size of ∼140 sheep until mid-autumn, when lambs reaching a target weight of 43.0 kg are gradually sent for slaughter.

It is now well-established that high frequency (> every 30 min.) data collection is required for accurate determination of sediment loss, especially for small catchments (Oliver and Rieger, 1988). This need has been assisted by the growing use of turbidity sensors based on the optical properties of water which cause light to be scattered or absorbed. The paired deployment of turbidity sensors and automatic water samplers can be used to develop robust ratings for converting turbidity time series into actual suspended sediment concentration (SSC) data (Wass and Leeks, 1999) and on this basis, many studies have used turbidity monitoring to estimate sediment loss (Goodwin et al., 2003; Walling et al., 2006; Minella et al., 2008; Stutter et al., 2017). For the work herein, turbidity sensors were calibrated monthly using a two-point procedure; 0 Formazine Nephelometric Units (FTU) using RO water and 124 FTU. Two sets of sensors are maintained meaning that all sensors are continuously replaced at the frequency of re-calibration. Flow-proportional automatic water samples collected at the flumes were filtered gravimetrically (using known sample volumes) for suspended sediment concentrations in the laboratory, using pre-weighed, dried glass fiber filter papers (1.2 μm pore size, Whatman GFC) which were dried at 105 °C for 1 h and re-weighed to determine sediment mass (Peukert et al., 2014). Given the marked change in the relationship between SSC-turbidity observed in response to the scheduled plough and reseeds on the blue and red treatments of the NWFP, two ratings were developed and applied. The first (Eq. 1), was used for the baseline (before the scheduled ploughing and grass re-seeds) and thereafter, post the end of the first winter drainage period following each scheduled re-seed, when grass cover had fully recovered. The second (Eq. 2), was applied to the turbidity time series for the period from each scheduled plough till the end of the ensuing winter drainage period (end of the following March):

(1) SSC = 1.1804 * NTU + 0.0472 (r^2^ = 0.75)

(2) SSC = 0.7664 * NTU + 5.7116 (r^2^ = 0.91)

**Fig. 3:**
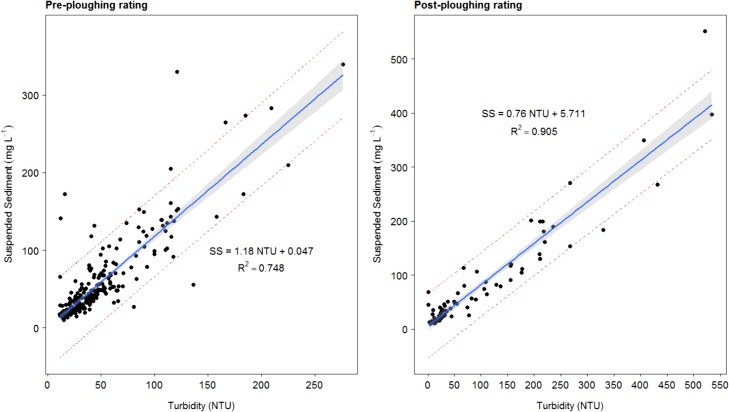
Suspended sediment concentration-turbidity ratings.

Each suspended sediment concentration–turbidity rating was constructed with 95 % prediction intervals and confidence limits (Fig. 3), to estimate the uncertainty for the sediment loss predictions which, in turn, covered the period 1/10/2012 – 31/03/2016. This time period spanned the three phases of scheduled plough and reseeds on the NWFP (2013–2015, inclusive; Fig. 2).

### 2.4 Use of the instrumented North Wyke Farm Platform to provide mechanistic understanding on sediment loss

In addition to providing an opportunity to measure sediment delivery, for comparison against MBSDR, the data yielded by the NWFP provided an opportunity to assess key controls on sediment loss. Recent work reported by Pulley and Collins (2019) examined the correlations between suspended sediment loss measured at the 15 flumes on the NWFP and a range of potential controls (Table 2). Critically, sediment export from the fields was strongly correlated with catchment area (i.e., hydrologically-isolated fields), but not with either percentage of the total catchment area damaged by soil poaching or the area of poached soil. On the basis of field observations, the specific mechanism identified for soil erosion and sediment loss was raindrop-impacted saturation-excess overland flow and this was observed to act field-wide rather than in specific within-field locations.

**Table 2:** Pearson correlation coefficients between potential controlling factors and sediment data on the NWFP (from Pulley and Collins, 2019); bold values are significant (p < 0.05).

	Rainfall	Percent time with animals	Rising limb water flux	Falling limb water flux	Baseflow water flux	Total water flux	Percentage time rising	Percentage time falling	Rainfall reaching outlet	Damaged soil area	Percent of soil area damaged	Water yield	Maximum flow rate	Mean SSC	Sediment flux	Sediment yield	Mean slope	Max flow accumulation
Area	−0.26	−0.25	**0.951**	**0.882**	**0.865**	**0.925**	**0.86**	**0.549**	0.329	**0.683**	**0.611**	0.117	**0.963**	0.202	**0.751**	0.031	0.244	**0.549**
Rainfall		0.225	−0.24	−0.21	−0.28	−0.24	−0.11	−0.06	−0.12	−0.23	−0.21	0.143	−0.27	**0.522**	0.156	**0.524**	**−0.52**	0.006
Percent of time with animals			−0.14	−0.17	−0.33	−0.21	−0.09	−0.10	−0.13	**0.641**	**0.692**	−0.06	−0.17	0.192	0.143	0.233	−0.08	−0.283
Rising limb water flux				**0.962**	**0.879**	**0.977**	**0.918**	**0.649**	0.489	**0.657**	**0.547**	0.21	**0.963**	0.242	**0.821**	0.159	0.286	**0.520**
Falling limb water flux					**0.924**	**0.991**	**0.93**	**0.783**	0.652	**0.657**	**0.522**	0.341	**0.883**	0.182	**0.733**	0.144	0.215	0.509
Baseflow water flux						**0.952**	**0.848**	**0.677**	**0.636**	**0.574**	0.457	0.374	**0.798**	0.109	**0.58**	−0.01	0.169	0.488
Total water flux							**0.926**	**0.724**	**0.604**	**0.651**	**0.527**	0.311	**0.911**	0.189	**0.742**	0.112	0.234	**0.520**
Percentage time rising								**0.853**	**0.700**	**0.539**	0.444	**0.535**	**0.857**	0.41	**0.824**	0.379	0.053	0.494
Percentage time falling									**0.863**	0.432	0.323	**0.71**	**0.549**	0.246	**0.521**	0.345	−0.06	0.320
Rainfall reaching outlet										0.243	0.131	**0.842**	0.327	0.252	0.347	0.457	−0.12	0.166
Damaged soil area											**0.968**	−0.05	**0.697**	−0.05	0.373	−0.07	0.222	**0.617**
Percent of soil area damaged												−0.06	**0.617**	−0.04	0.315	−0.07	0.142	**0.671**
Water yield													0.066	0.463	0.277	**0.564**	−0.45	0.089
Maximum flow rate														0.217	**0.773**	0.10	0.366	**0.574**
Mean SSC															**0.625**	**0.907**	**−0.53**	0.189
Sediment flux																**0.579**	−0.03	0.407
Sediment yield																	−0.48	0.079
Mean slope																		−0.052

### 2.5 Scaling out with modelling for estimating the sediment loss ‘gap’ due to grazing ruminant farming beyond the instrumented farm platform

Scaling out beyond heavily-instrumented experimental farm platforms testing best management practice requires modelling. Here, the intention was to scale out a comparison of the potential for reducing the sediment loss ‘gap’ under business-as-usual farm management using on-farm interventions likely to be shortlisted either by current advisory inspections or those preselected on the basis of the new mechanistic understanding on sediment loss from the NWFP provided by Pulley and Collins (2019). Modelled sediment delivery to rivers from agricultural land was based on predictions from the PSYCHIC (Phosphorus and Sediment Yield CHaracterisation In Catchments) process-based model (Collins et al., 2007; Davison et al., 2008) which has been successfully evaluated using both local and strategic scale data (Stromqvist et al., 2008; Collins and Anthony, 2008; Collins et al., 2008, 2009; Comber et al., 2013; Collins and Zhang, 2016; Collins et al., 2016; Zhang et al., 2017). The efficiency of the subsurface flow pathway for sediment delivery, represented in the model, was modified using recent work reported by Zhang et al. (2016) which used farm surveys to reduce default model drain delivery efficiency using survey returns on drain maintenance.

**Fig. 4:**
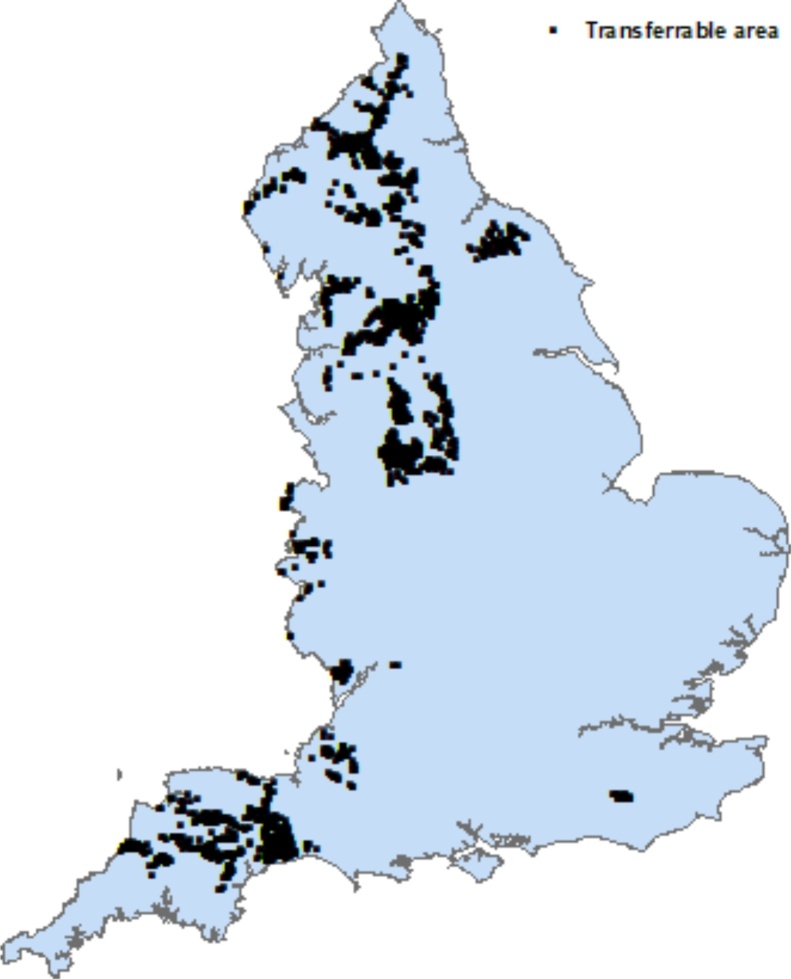
The transferable areas (1843 km^2^) for which the NWFP is representative in terms of key environmental factors and farm system.

To map the spatial domains across England, to which science from the NWFP applies, national scale data layers on key environmental and farm management characteristics were used. Long-term (1961–1990) average annual rainfall (AAR) across England (Barrow et al., 1993) has previously been used (e.g., Zhang et al., 2017) to map the spatial extent of six rainfall bands (<600 mm, 600−700 mm, 700−900 mm, 900−1200 mm, 120−1500 mm, >1500 mm). Since the available long-term rainfall record (1982–2016) at North Wyke indicated an average annual total of 1050 mm, the scaling out was based on the spatial extent of the 900−1200 mm band. The dominant soil series of the NWFP are represented by HOST (Hydrology of Soil Types; Boorman et al., 1995) classes 21 (Halstow) and 24 (Hallsworth) and the extent of these across England was mapped using a national database (NATMAP1000; National Soil Resources Institute, Cranfield University, UK). Since the average field slopes on the NWFP range between 2.5–6.9°, a national layer depicting slopes at risk of soil erosion (3−7°, Defra, 2009) was used to map coincidence with the rainfall and soil type classes above. Farm systems in England are classified into Robust Farm Types (RFTs; Defra, 2010) using the dominant five-year averaged standard outputs (the total value of the output generated by any single farm enterprise; combining the value of main, e.g. meat, and secondary, e.g. wool, products, minus replacement costs) calculated from the June Agricultural Survey (JAS). The spatial extent of lowland grazing livestock and less favoured area (LFA) grazing livestock farms across England was mapped using 2016 JAS data grouped by RFT. These data were summarized at Water Framework Directive (WFD) waterbody scale, and only those waterbodies wherein these farm systems accounted for >50 % of the utilized agricultural area (UAA) were included in the spatial domain for scaling out. Representative grazing ruminant model farms in the spatial domains represented by the intersection of the rainfall, soil, slope and farming system categories described above (amounting to 1843 km^2^ of England; Fig. 4) were built using the JAS 2016 data on livestock numbers and categories and crop areas. Critically, business-as-usual uptake of best management practice on the model farms, due to regulation, incentivization and advice, was also included in model farm construction. For the NWFP, the management information was provided by the farm manager. For the grazing ruminant farms across England in the spatial domains represented by the NWFP, typical practices and uptake rates under business-as-usual were extracted from a number of sources, including the Defra User Guide (Newell-Price et al., 2011), the Defra Farm Practices Survey (Defra, 2019), recent survey work in sentinel research catchments in the UK (Collins et al., 2016) and recent survey information on uptake rates of pollution control measures provided by the national Catchment Sensitive Farming initiative in England (Zhang et al., 2017).

### 2.6 Simulating the potential benefits of sediment management interventions on grazing ruminant farms

On-farm interventions for managing agricultural water pollution in England are currently delivered through a mix of regulation, incentivization and advice and collectively, these result in farm management under business-as-usual (Collins and Zhang, 2016). NWFP records were used to confirm current practice on the three farmlets (Table 3). Policy-preferred interventions (Newell-Price et al., 2011), and specifically those for controlling sediment loss, were selected for running modelling scenarios. Here, the intention was to compare one scenario representing the measures frequently recommended on the basis of visual audits of water quality problems on grazing ruminant farms and another, based on a group of measures shortlisted using the new mechanistic understanding, but still selected from recommended options. Visual appraisals typically result in recommendations targeting the visually obvious areas of erosion due to poaching including those around feeder areas, troughs or gateways or along fence lines especially at riparian edges along watercourses. In contrast, the mechanistic understanding from the NWFP clearly suggests that interventions with the potential to manage soils field-wide, rather than at sub-field level are more relevant for reducing sediment loss on the heavy soils in question. Two sets of on-farm interventions were thereby selected on this basis (Table 4). The technically feasible impacts, relative to business-as-usual, of these two management scenarios, both on the NWFP and on grazing ruminant farms in similar environmental settings more strategically across England, was modelled using an established framework (Gooday et al., 2014; Collins et al., 2016; Collins and Zhang, 2016; Zhang et al., 2017) which takes account of current implementation under business-as-usual and calculates the outcomes of increased uptake of interventions, assuming multiplicative interactions between those interventions. Both scenarios assumed 100 % implementation of the shortlisted interventions to assess maximum potential impact.

**Table 3:** Existing on-farm measures implemented on the NWFP.

Make use of improved genetic resources in livestock
Fertiliser spreader calibration
Use a fertiliser recommendation system
Integrate fertiliser and manure nutrient supply
Do not apply manufactured fertiliser to high-risk areas
Avoid spreading manufactured fertiliser to fields at high-risk times
Use manufactured fertiliser placement technologies
Replace urea fertiliser to grassland with another form
Do not apply P fertilisers to high P index soils
Reduce field stocking rates when soils are wet
Construct troughs with concrete base
Manure Spreader Calibration
Do not apply manure to high-risk areas
Do not spread FYM to fields at high-risk times
Fence off rivers and streams from livestock
Calibration of sprayer
Fill/Mix/Clean sprayer in field
Avoid PPP application at high risk timings
Drift reduction methods

**Table 4:** The lists of on-farm interventions used to represent the two modelled scenarios for grazing ruminant farms.

Visually-based farm audit recommended interventions Move feeder rings at regular intervals
Construct troughs with a concrete base
Re-site gateways away from high risk areas
Farm track management
Establish riparian buffers

Mechanistically-based interventions
Reduce the length of the grazing season
Reduce field stocking rates when soils are wet
Locate out-wintered stock away from watercourses
Loosen compacted soil layers in grass fields
Use correctly-inflated low ground pressure tyres

## 3 Results and discussion

### 3.1 Exceedance of modern background sediment delivery to rivers under grazing ruminant farm business-as-usual management

Table 5 presents a summary of the suspended sediment loss measured on the NWFP between 1/10–2012 – 31/03/2016 and the corresponding compliance with, or exceedance of, MBSDR. For the permanent pasture treatment, total sediment loss over the monitoring period ranged between 0.65−0.78 t equating to 0.11−0.14 t ha^−1^ yr^−1^ and 4.44–5.38 t or 0.21−0.25 t ha^−1^ yr^−1^. Two of the five NWFP catchments under permanent pasture (green treatment; Figs. 1,2) exceeded TMBSDR but none exceeded MMBSDR. On the grass/clover reseeded mix treatment (blue treatment; Figs. 1,2), total sediment loss ranged between 1.09–1.32 t, equating to 0.19−0.23 t ha^−1^ yr^−1^ and 9.89–11.64 t or 0.43−0.51 t ha^−1^ yr^−1^. Three of the five catchments exceeded TMBSDR and one exceeded MMBSDR. On the treatment hosting the high sugar grass monoculture reseed (red treatment; Fig. 1,2), total sediment loss ranged between 0.55−0.66 t (0.10−0.13 t ha^−1^ yr^−1^) and 5.47–6.69 t (0.25−0.31 t ha^−1^ yr^−1^). Again, three of the five catchments under this land cover treatment exceeded TMBSDR and only one exceeded MMBSDR.

**Table 5:** Uncertainty ranges in measured sediment loss (October 2012 – March 2016) from the NWFP farmlets and corresponding exceedance of MBSDR.

Treatment / Flume*	95 % confidence limit range for total sediment loss (t)		95 % confidence limit range for specific sediment loss (t ha^−1^) 1		95 % confidence limit for annual specific sediment loss (t ha^−1^ yr^−1^) 2		Exceedance of MBSDR target^3^	
							TMBSDR	MMBSDR
Permanent pasture (green)								
4	6.31	7.82	0.73	0.91	0.23	0.28	Y	N
5	4.44	5.38	0.68	0.82	0.21	0.25	Y	N
6	1.83	2.30	0.47	0.60	0.15	0.18	N	N
12	0.65	0.78	0.37	0.44	0.11	0.14	N	N
13	0.86	1.02	0.49	0.58	0.15	0.18	N	N
Grass/clover mix (blue)								
7	1.50	3.60	0.58	1.39	0.21	0.50	Y	N
8	9.89	11.64	1.41	1.66	0.43	0.51	Y	Y
9	5.04	6.10	0.65	0.79	0.20	0.24	N	N
11	1.09	1.32	0.62	0.75	0.19	0.23	N	N
14	1.40	1.69	0.81	0.98	0.28	0.34	Y	N
High sugar monoculture (red)								
1	1.55	2.30	0.32	0.48	0.12	0.17	N	N
2	5.47	6.69	0.82	1.01	0.25	0.31	Y	N
3	5.68	7.01	0.86	1.06	0.26	0.33	Y	N
10	0.50	0.66	0.28	0.36	0.10	0.13	N	N
15	1.88	2.30	1.22	1.49	0.38	0.46	Y	Y

* Flume numbers shown on Fig. 1.

1 normalised by catchment hydrological area.

2 normalised by catchment hydrological area and duration of monitoring.

3 targets shown in Table 1.

**Fig. 5:**
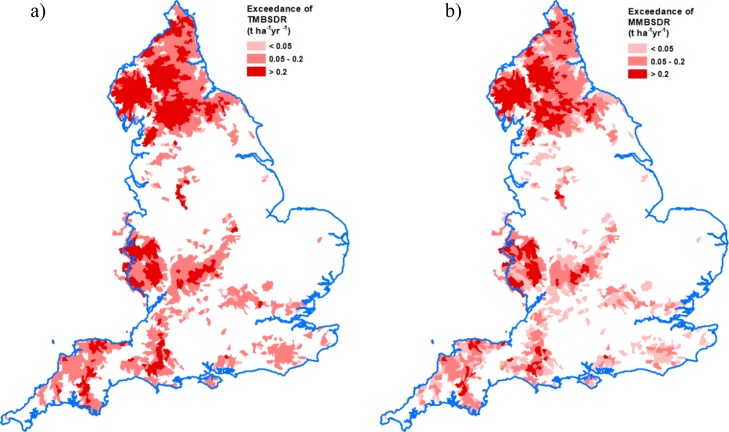
Exceedance of TMBSDR and MMBSDR to rivers.

The data in Table 5 show that exceedance of TMBSDR by the NWFP catchments was more prevalent on the two treatments (grass/clover mix and high sugar grass monoculture) which experienced scheduled plough and reseeds during (2013–2015, inclusive; Fig. 2) the monitoring period. Some exceedance of MMBSDR was only measured on the two treatments with grass reseeds. Such sward improvement operations are characteristically part of pasture management in the UK (Hopkins et al., 1990; Hopkins and Wilkins, 2006) but run the risk of elevating soil loss if soil moisture content is high and wet weather is experienced in the immediate period post drilling due to the exposure of bare ground and subsequently soil surfaces with an emerging sward cover. Soils on the NWFP are primarily land capability (Bibby et al., 1982) class 4, but also 5, both of which indicate severe soil wetness driven limitations for land cover types and associated farming operations. Fig. 5 shows that the spatial extent of exceedance of TMBSDR and MMBSDR due to losses from the agricultural sector coincides with the transferable areas for NWFP science (Fig. 4). Across England, the exceedance of MBSDR due to losses from agriculture can be >0.2 t ha^−1^ yr^−1^ in some areas, illustrating the need for improved best management for closing the loss gap, and thereby the relevance of testing the potential added benefits of alternative management futures, over and above business-as-usual, based on the new mechanistic understanding of sediment loss from the NWFP intensive monitoring (Pulley and Collins, 2019).

### 3.2 Evaluation of modelled sediment loss from grazing ruminant farms under business-as-usual

**Fig. 6:**
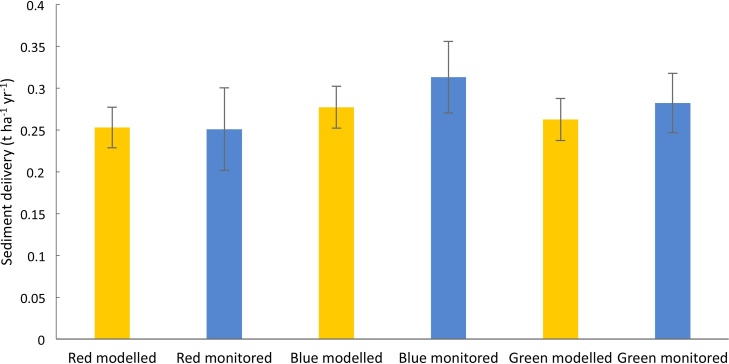
Comparison of modelled and measured sediment delivery to rivers on the NWFP, showing 95 % confidence limits.

**Fig. 7:**
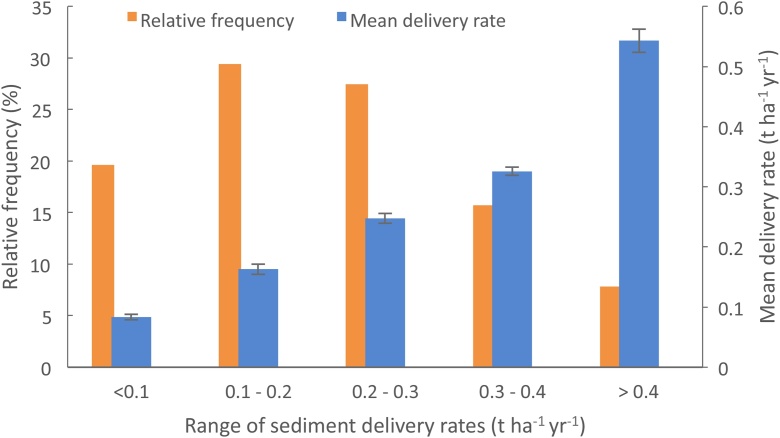
Histogram of modelled sediment delivery to rivers (showing 95 % confidence limits) from land used for grazing ruminant farming under business-as-usual in areas with similar environmental settings to the NWFP.

Fig. 6 compares the modelled and measured sediment loss on the NWFP. This figure illustrates good agreement between the heavily-instrumented farm monitoring data and the PSYCHIC-based predictions of sediment loss under business-as-usual. Heavily-instrumented grazing ruminant farms do not exist more strategically across England, meaning that evaluation of the modelled sediment losses under business-as-usual in the areas represented by the NWFP is reliant on comparison with published data. A histogram of the modelled business-as-usual sediment losses from grazing ruminant farms across England is provided in Fig. 7. The modelled sediment losses range from 0.05 t ha^−1^ yr^−1^ to 0.58 t ha^−1^ yr^−1^, with losses of <0.1 t ha^−1^ yr^-1^ predicted for 20 % of the model farms, compared with 0.1−0.2 t ha^−1^ yr^−1^for 29 %, 0.2−0.3 t ha^−1^ yr^−1^for 27 %, 0.3−0.4 t ha^−1^ yr^−1^for 16 % and >0.4 t ha^−1^ yr^−1^ for 8%. On the basis of combining sediment source fingerprinting data for 71 sub-catchments across England (e.g., Collins et al., 2010), information on spatially-extrapolated suspended sediment yields (Cooper et al., 2008) and different landscape retention factors to convert the estimates to sediment delivery to rivers, Evans et al. (2017) reported that current sediment loss from farmed grassland on heavy soils ranges from between 0.24−0.63 t ha^−1^ yr^-1^ and 0.28−0.73 t ha^−1^ yr^−1^. Published suspended sediment yields for pasture dominated catchments in England cover a wide range (Evans, 2006), with examples including 0.01 t ha^−1^ yr^-1^ (Oxley, 1974), 0.09−0.19 t ha^−1^ yr^-1^ (Foster and Lees, 1999) and 0.01-1.6 t ha^−1^ yr^-1^ (Walling, 1990). These values, *sensu stricto*, do not represent sediment delivery to rivers, but instead, net sediment export at landscape scale, but nonetheless are useful for sanity checking the modelled sediment loss rates in Fig. 7. Collectively, these published sources of data indicate that the PSYCHIC-based predictions of business-as-usual sediment delivery to rivers from land used by grazing ruminant farming across England are consistent with current evidence.

### 3.3 Potential for closing the sediment loss ‘gap’ using on-farm interventions likely to be selected by visual farm audits or hydro-sedimentological mechanistic understanding

**Fig. 8:**
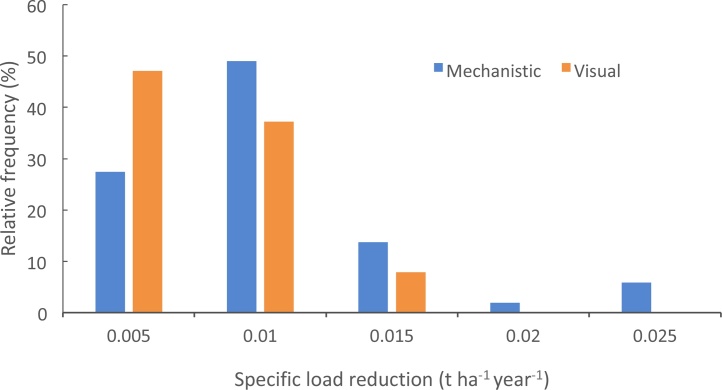
Histogram of modelled absolute reductions in sediment delivery to rivers delivered by the two scenarios on farms exceeding MBSDR.

Table 6 presents the percentage reductions in sediment delivery for the two intervention scenarios for the three farmlets on the NWFP and for grazing ruminant farms more strategically across England but in similar environmental settings as the instrumented farm platform. Mechanistically-selected interventions would reduce business-as-usual sediment delivery by 5.0 % on the NWFP compared with an average of 3.5 % on grazing ruminant farms more strategically across England in similar environment settings. The corresponding respective reductions predicted for the scenario using interventions typically recommended by visual farm audits were predicted to be 2.1 % and 2.3 %. Neither future management scenario improved the current compliance (Table 5) with either MBSDR rates on the NWFP. Here, the absolute reductions in business-as-usual sediment delivery were predicted to be 0.007 t ha^−1^ yr^-1^ and 0.018 t ha^−1^ yr^-1^ for the visually- and mechanistically-based intervention scenarios, respectively. In the case of the modelled grazing ruminant farms across England in similar environmental settings to the NWFP, 57 % were predicted, under business-as-usual, to have sediment loss exceeding TMBSDR and 31 % exceeding MMBSDR. Both intervention scenarios were predicted to reduce the proportion of farms exceeding TMBSDR to 29 %. In the case of exceedance of MMBSDR, the mechanistically-based scenario reduced the proportion of farms predicted to have excess sediment delivery from 31 % to 29 %. The visually-based scenario delivered no reduction in the proportion of grazing livestock farms exceeding MMBSDR. In terms of the absolute magnitude of the reductions in sediment delivery, under the two scenarios, Fig. 8 shows the frequency distributions of the modelled absolute reductions in business-as-usual sediment delivery to rivers from the grazing livestock farms across England exceeding MBSDR. The mechanistically-based scenario was predicted to deliver a maximum reduction of 0.023 t ha^−1^ yr^−1^ whereas the corresponding maximum reduction with the visually-based scenario was 0.014 t ha^−1^ yr^−1^. These results point clearly to the need to consider more severe land cover change options on those grazing ruminant farms exceeding MBSDR, since even increased uptake of the field wide measures selected on the basis of the mechanistic understanding of soil loss on the NWFP, is predicted to deliver limited benefits above and beyond what is currently being achieved as a result of business-as-usual farm management.

**Table 6:** Summary results for the predicted relative reductions in business-as-usual sediment delivery to rivers associated with the two intervention scenarios for the NWFP and grazing ruminant farms in areas across England with matching soils, rainfall and slopes.

	Scenario	
NWFP	Visually-based audit	Mechanistically-based
Permanent pasture (green)	2.1	5.0
Grass/clover mix (blue)	2.1	5.0
High sugar grass monoculture (red)	2.1	5.0
		
Grazing ruminant farms in areas matching the NWFP	2.3	3.5

### 3.4 Implications

Visual farm audits and walkovers (frequently one-off) remain at the core of on-site appraisals for recommending interventions for tackling diffuse water pollution on farms in England. The new hydro-sedimentological mechanistic evidence from the NWFP, however, caveats against relying on the visual appraisal approach in isolation. Instead, it suggests, that in parallel to such traditional auditing, scientific evidence on fundamental mechanisms driving the externality in question (elevated sediment loss in this case) should be considered carefully and used to help select interventions from recommended lists. This conclusion provides further support of the need to assemble or draw upon robust mechanistic evidence in planning diffuse sediment pollution mitigation programmes reported by Biddulph et al. (2017) as part of the UK Demonstration Test Catchment (DTC) programme. Visual appraisals typically result in localized areas of fields undergoing poaching such as around feeder rings, troughs, fence lines or gateways being earmarked for intervention, but given that such areas are spatially-limited, their contribution to total sediment loss from grazed fields is limited, even where those fields have good sward cover. Previous work combining sediment source fingerprinting and a dual-signature particle tracking method reached a similar conclusion, albeit in an upland setting in northern-western England (Collins et al., 2013). Although neither future management scenario closed the sediment loss gap under business-as-usual, the mechanistically-based scenario performed marginally better than the alternative framed to represent the measures likely to be recommended to a farmer during visual audits of erosion problems on grazing ruminant farms.

The modelling results clearly indicate that grazing ruminant farming futures based on either of the scenarios will not close excess sediment loss, relative to modern background, where such a gap exists (57 % of the modelled farms using TMBSDR and 31 % using MMBSDR). Recommended on-farm interventions for protecting water quality in England are currently supported by a mix of regulation, incentivization and advice and the interplay of these policy instruments is responsible for farm best management under business-as-usual. The modelling of the two scenarios assumed that the interplay between the on-farm measures in either scenario is multiplicative rather than additive, meaning that the predictions of impact are cautious. Here, it is useful to acknowledge that wide-ranging experimental or empirical evidence on the interactions between targeted measures, currently supported by policy instruments, remains scant and this is clearly an evidence gap that instrumented farm platforms such as the NWFP can address using carefully selected experiments. Any modelling of on-farm interventions is reliant on a combination of the limited experimental evidence base and elicitation of expert judgement on efficacy and Table S2 summarizes current understanding on the efficacy of the interventions modelled in this work.

Under the current Countryside Stewardship agri-environment scheme in England, farmers in higher tier areas have the option to consider the creation of wood pasture (option WD6; https://www.gov.uk/countryside-stewardship-grants/creation-of-wood-pasture-wd6). The area payment for this option is £409 ha^−1^ and payments for each agreement last for 10 rather than the normal five years. The option can be targeted at sites which were previously wood pasture or where any new planting extends, links or buffers existing wood pasture or priority woodland habitats. A snapshot of scheme option uptake rates in 2016 suggests that there were only 24 such agreements in place, compared with for example, 3717 for woodland improvement (option WD2). This suggests there remains significant opportunity for increasing uptake rates. In the UK, high level policy targets for woodland creation have been specified (e.g., Defra, 2013) in the context of the current low proportion (13 %) of woodland cover (FAO, 2015). Woodland creation is, however, debated rigorously in the context of conflicting high-level food production and climate change mitigation policy goals (Duckett et al., 2016; Burton et al., 2018a) and woodland planting faces many barriers including low acceptability among farmers (Duckett et al., 2016; Burton et al., 2018b). Lack of advice has also been underscored as a barrier (Lawrence and Dandy, 2013). Regardless, the fact remains that targeting tree planting, including on land used by grazing ruminants, would improve protection against erosion by modifying hydrology and dampening runoff and flood peaks (Thomas and Nisbet, 2007; Dixon et al., 2016).

In the context of the UK’s decision to depart the European Union, a new Environmental Land Management (ELM) scheme will be launched with a national pilot in 2021, leading to full implementation in late 2024 (HM Government, 2018). The current policy vision suggests that the new ELM scheme will comprise three tiers. Tier 1 will incentivize environmentally friendly farming by supporting best management practices at scale. Tier 2 will support land managers to deliver locally-targeted environmental outcomes, especially on the basis of collaboration between farmers. Finally, Tier 3 will deliver landscape scale land use change for the most ambitious environmental targets. The findings of our research are highly relevant to the new ELM scheme and suggest that some land cover change will be necessary to address the principal mechanism for sediment delivery from the study soils and grazing ruminant farming systems, since conventional interventions selected either on the basis of visual audits or mechanistic understanding have the potential to deliver very limited reductions in addition to business-as-usual. The sediment pollution gap will therefore not be closed using existing recommended interventions. Clearly, the role of credible, trusted and consistent advice (Dampney et al., 2001; Prager and Thomson, 2014; Rose et al., 2019) will be crucial for the success of the new ELM scheme and on the basis of the work reported in this paper, advisors should be trained in combining the use of traditional walkover visual appraisals with consideration of available mechanistic evidence for sediment loss or indeed additional externalities arising from modern farming methods. Here, heavily-instrumented farm platforms like the NWFP have a crucial role to play in providing long-term robust mechanistic evidence which can be scaled out using models, as demonstrated herein.

## 4 Conclusion

The work reported herein illustrates that two shortlists of recommended on-farm measures selected to capture either visual audits or to address mechanistic understanding for soil loss and sediment delivery on the soils in question, are unable to close the exceedance or sediment loss gap. More severe land use change is therefore required to close exceedance of MBSDR on the farms in question. Future work will examine the trade-offs at both farm and landscape scale associated with different management scenarios, including those founded on mechanistic understanding of plant-soil-water interactions in agriculture. The intention will continue to be to appraise the value of using mechanistic understanding to refine lists of measures recommended by conventional visual farm audits targeting agricultural sustainability and water pollution problems. In terms of impact on the sediment water pollution gap, mechanistically-defined sets of interventions most likely represent the middle ground between visually-based interventions and the more severe option of land use conversion (e.g., to woodland), which continues to be unpopular with many farmers.

## CRediT authorship contribution statement

**A.L. Collins:** Funding acquisition, Supervison, Conceptulisation, Methodology, Formal analysis, Writing - original draft, Writing - review & editing. **Y. Zhang:** Formal analysis. **H.R. Upadhayay:** Formal analysis. **S. Pulley:** Formal analysis. **S.J. Granger:** Formal analysis. **P. Harris:** Data curation. **H. Sint:** Data curation. **B. Griffith:** Investigation.

## Declaration of Competing Interest

The authors declare that they have no known competing financial interests or personal relationships that could have appeared to influence the work reported in this paper.
